# Supplementary material for: The SAS chromatin-remodeling complex mediates inflorescence-specific chromatin accessibility for transcription factor binding
Source: Nucleic Acids Res. 2025 Apr 29;53(8):gkaf316. doi: 10.1093/nar/gkaf316 (PMC12038394; doi:10.1093/nar/gkaf316)
Supplement: gkaf316_Supplemental_Files [file gkaf316_supplemental_files.zip › Supplementary Figure_S1-S9.pdf]

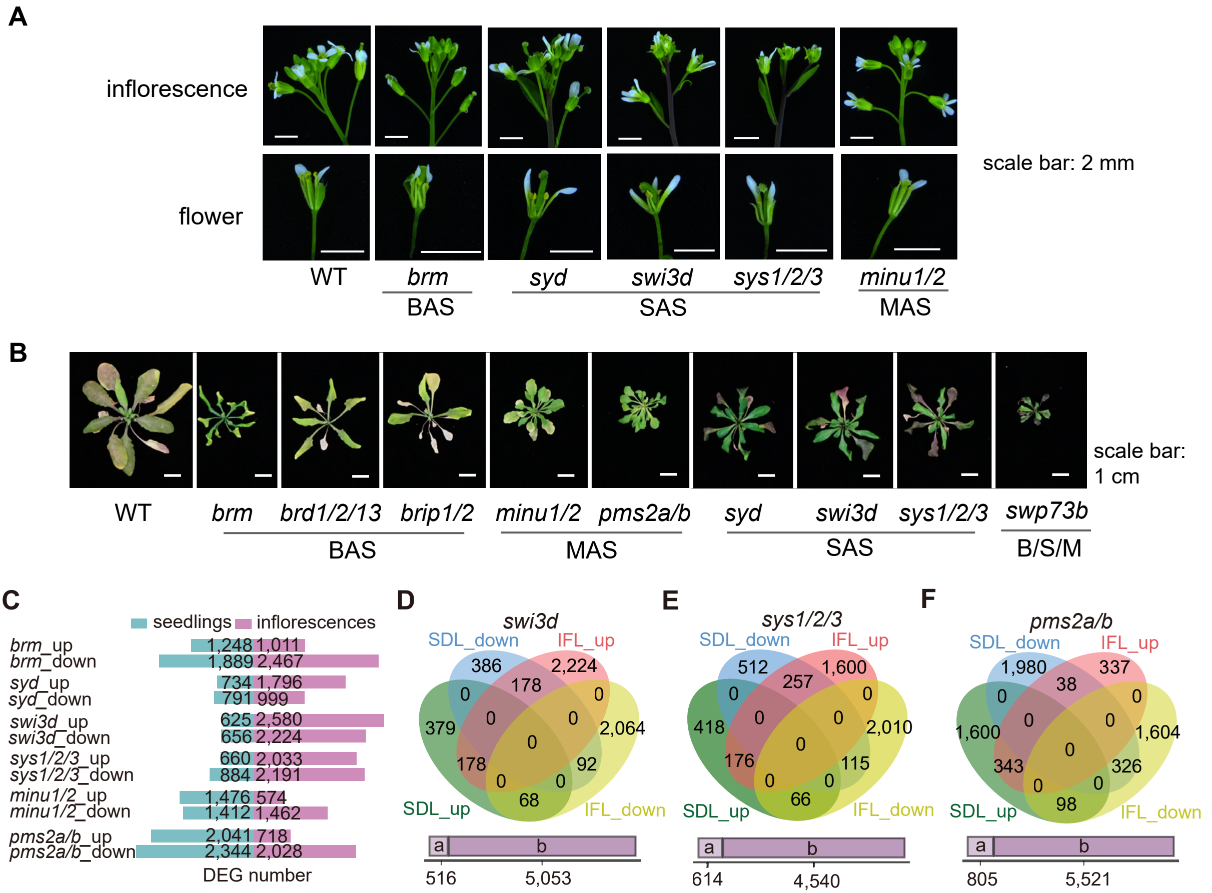

Supplementary Figure S1. Inflorescence phenotype and different gene expression analysis in SWI/SNF mutants. (**A**) Photographs of inflorescences and flowers from 5~6-week-old SWI/SNF mutants. To show the inner stamens and pistil of a flower, some sepals and petals are removed in the second row of pictures. (**B**) Images of rosette leaves from 8-week-old SWI/SNF mutants. To show the anthocyanin accumulation in rosette leaves, shoots and inflorescences were removed from the plant. **C)** The number of up-regulated and down-regulated DEGs in seedlings of inflorescences of SWI/SNF mutants, as determined by RNA-seq. (**D-F**) Venn diagrams showing the overlap of up-regulated DEGs and down-regulated DEGs in seedlings and inflorescences of *swi3d* (**D**), *sys1/2/3* (**E**), and *pms2a/b* (**F**). The purple bar below the venn diagram marked by ‘a’ and ‘b’ represent genes shared by other sets and genes specifically belonging to one set of the venn diagrams, respectively.

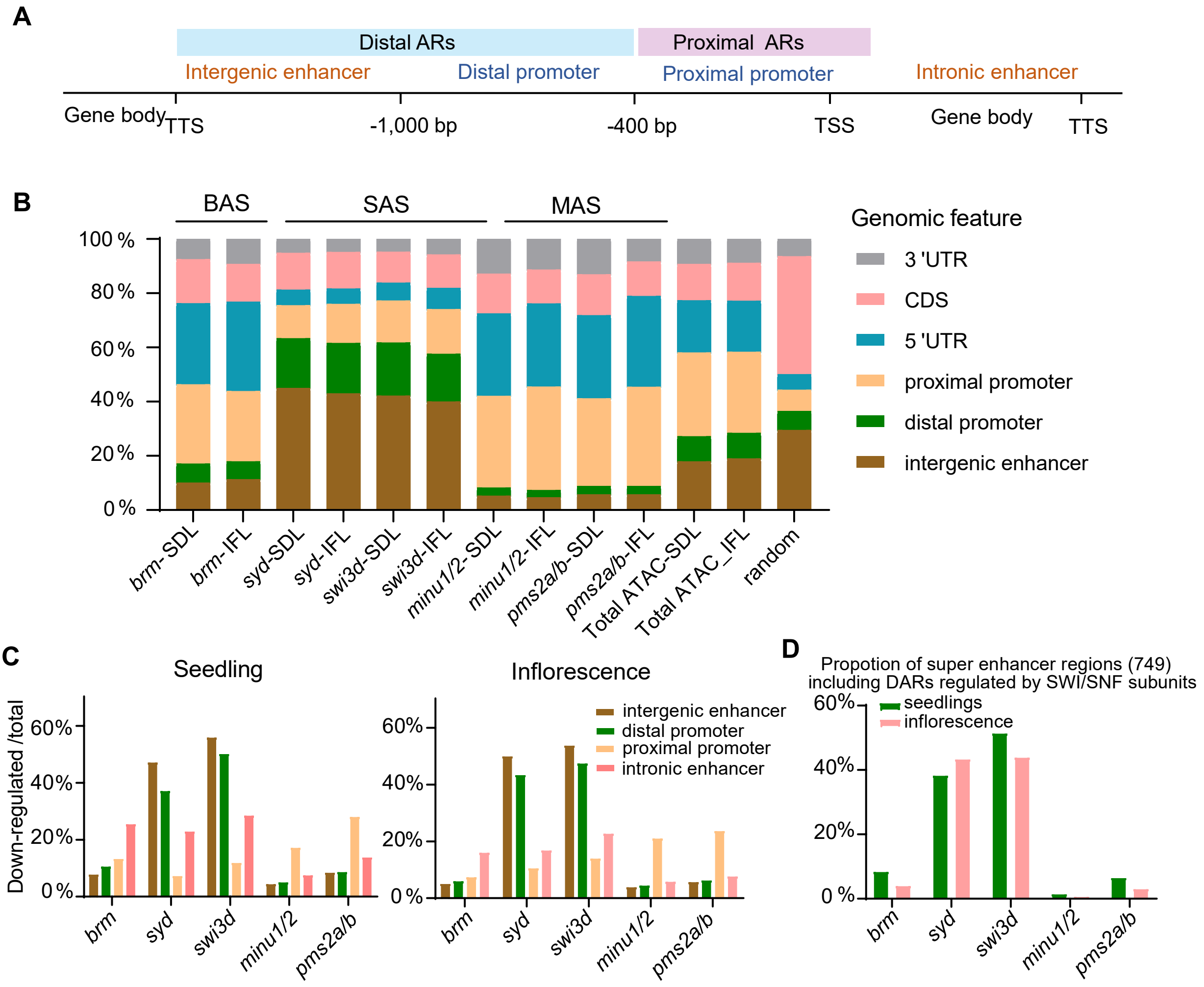

Supplementary Figure S2. Proximal and distal accessible regions regulated by SWI/SNF complexes in seedlings and in inflorescences. (**A**) The illustration of specified genomic features used for the peak annotation. ARs, accessible regions. (**B**) Proportion of down-regulated differentially accessible regions annotated to specified features to the total down-regulated differentially accessible regions in seedlings or inflorescences of the indicated SWI/SNF mutants. The proportion of accessible regions annotated to specified genomic features to total accessible regions identified by ATAC-seq in the seedlings and inflorescences of wild-type plants is also presented. Random: 8,000 random genomic sites. DARs, differentially accessible regions. (**C**) Proportion of downregulated differentially accessible regions in each SWI/SNF mutant to the total accessible regions annotated to specified genomic features in seedlings and in inflorescences. (**D**) The proportion of super enhancers including accessible regions regulated by SWI/SNF components in seedlings and in inflorescences to total super enhancers identified in a previous study (Zhao et al., 2022).

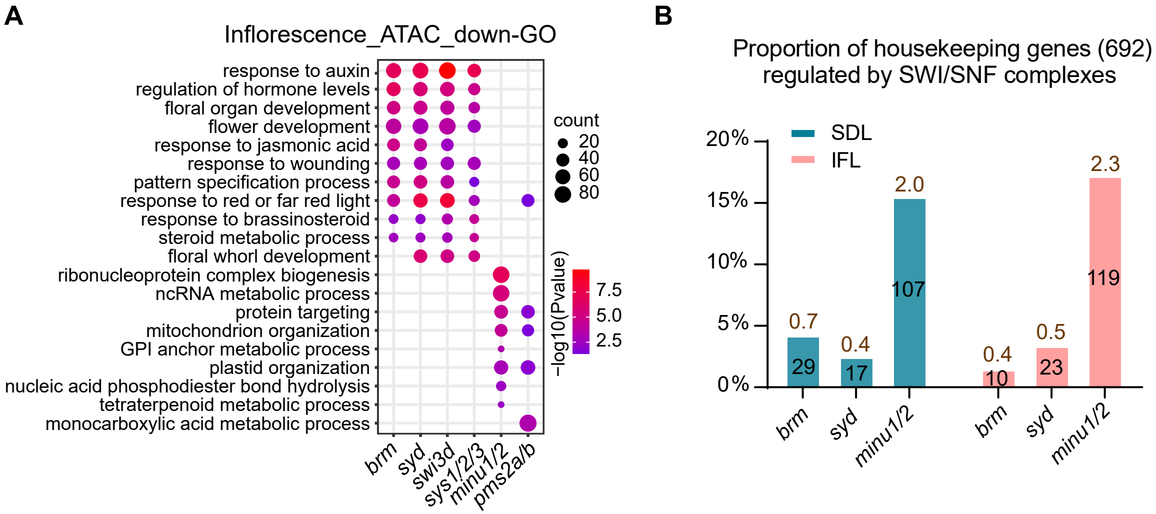

Supplementary Figure S3. Characteristics of genes with down-regulated accessibility in inflorescences of SWI/SNF mutants. (**A**) GO enrichment of genes with down-regulated accessibility in inflorescences of SWI/SNF mutants. Bubble plots show the enriched GO terms in genes with down-regulated accessibility in inflorescences of SWI/SNF muants. (**B**) The propotions of housekeeping genes (identified by Cheng et al., 2017) showing down-regulated accessibility in seedlings (SDL) and inflorescences (IFL) of the SWI/SNF mutants. The number of overlaping genes are marked on the bars with black font. The values marked by the brown font above the bars represent the Representative Factors (the number of overlaping genes divided by the expected number of overlaping genes drawn from two independent groups).

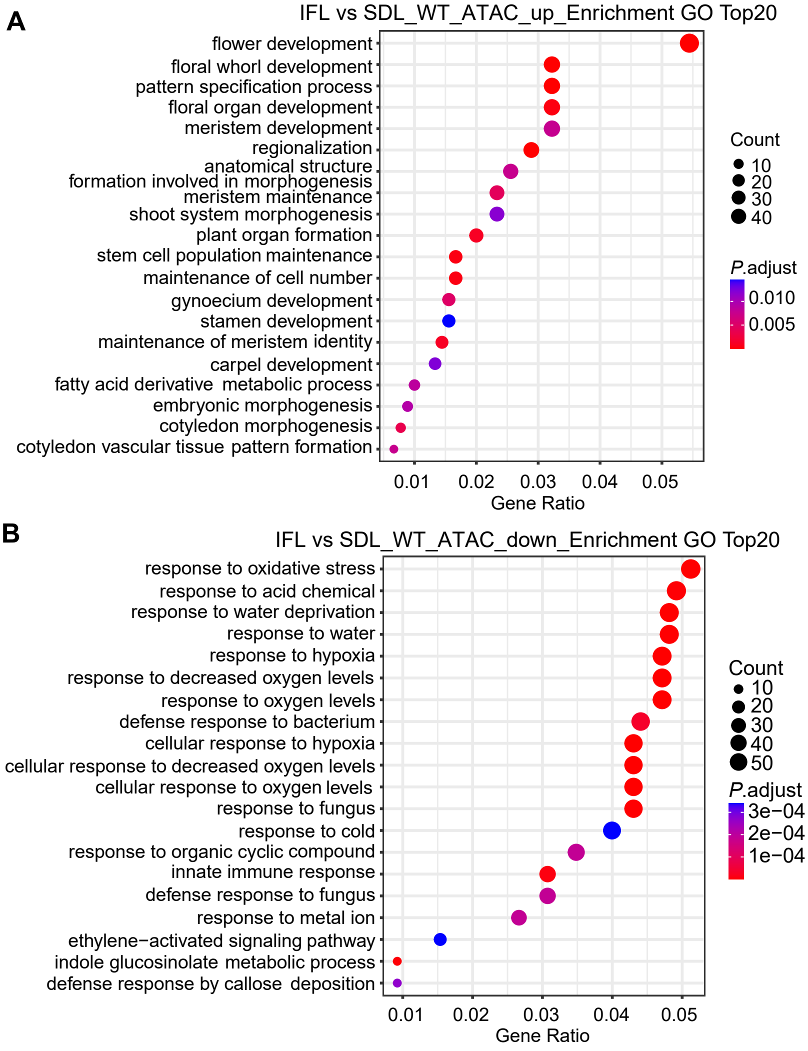

Supplementary Figure S4. GO enrichment of seedling-specific and inflorescence-specific accessible genes. (**A, B**) Bubble plots showing the top 20 enriched GO terms in specific accessible genes in inflorescences (**A**) and seedlings (**B**) of wild-type plants.

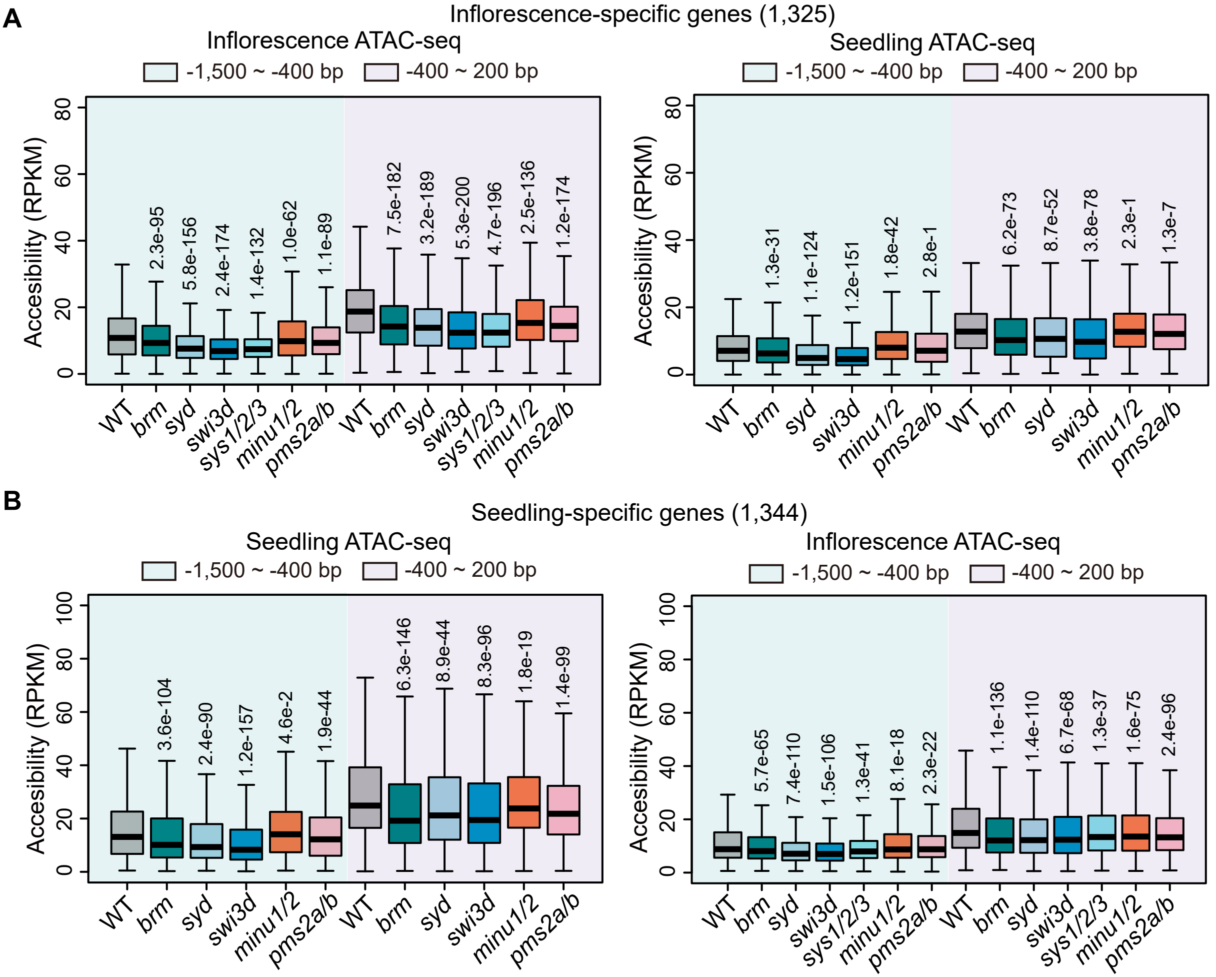

Supplementary Figure S5. Effects of SWI/SNF subunit mutations on chromatin accessibility at inflorescence-specific accessible genes and seedling-specific genes. (**A, B**) Boxplots showing the chromatin accessibility levels in seedlings and inflorescences of the wild type and SWI/SNF mutants at the 400-1,500 upstream region of TSS and at the -400-200 bp TSS-flanking region. Analysis was conducted separately for inflorescence-specific genes (**A**) and seedling-specific genes (**B**). The center lines and box edges of the box plots represents medians and the interquartile range (IQR), respectively. Whiskers extend within 1.5 times the IQR. *P* values were determined by two-tailed Wilcoxon signed rank test between the mutants and the wild-type control in each group.

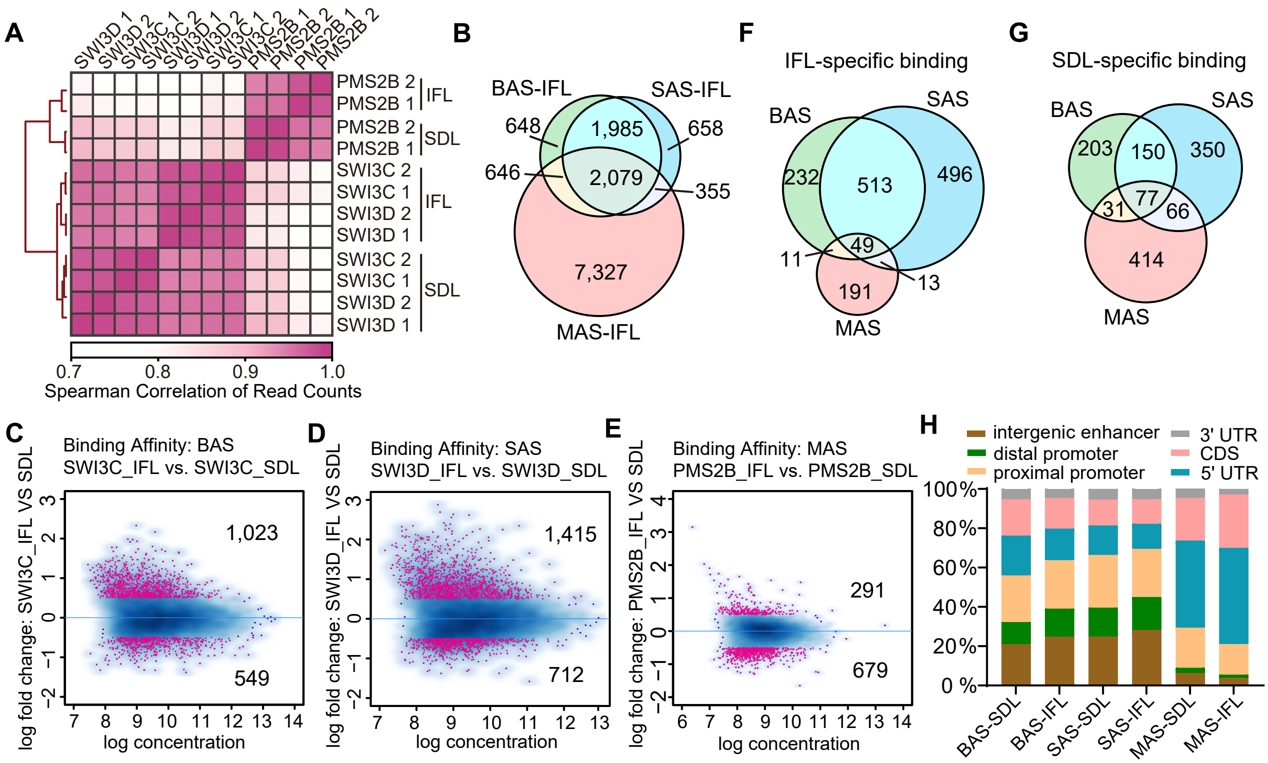

Supplementary Figure S6. Comparison of the binding regions of BAS, SAS and MAS in seedlings and inflorescences. (**A**) Heat map showing the pairwise Spearman correlation coefficients based on the ChIP-seq read counts of the two replicates of BAS (SWI3C), SAS (SWI3D), and MAS (PMS2B) subunits in seedlings or in inflorescences. (**B**) Venn diagram illustrating the overlap of the genes bound by BAS, SAS and MAS in inflorescences. (**C-E)** MA plots showing the up-regulated and down-regulated binding peaks of the BAS (**C**), SAS (**D**) and MAS (**E**) subunits in inflorescences compared to seedlings. The red points represent significant changed peaks with FDR<0.05 and $|\log_2 (\text{fold change})| >= 0.5$. (**F, G)** Venn diagrams showing the overlap of genes specifically bound by BAS, SAS and MAS in inflorescences (**F**) and in seedlings (**G**). (**H**) The proportion of regions bound by BAS, SAS and MAS annotated to specified chromatin features in seedlings and in inflorescences. Random: 8,000 random genomic sites.

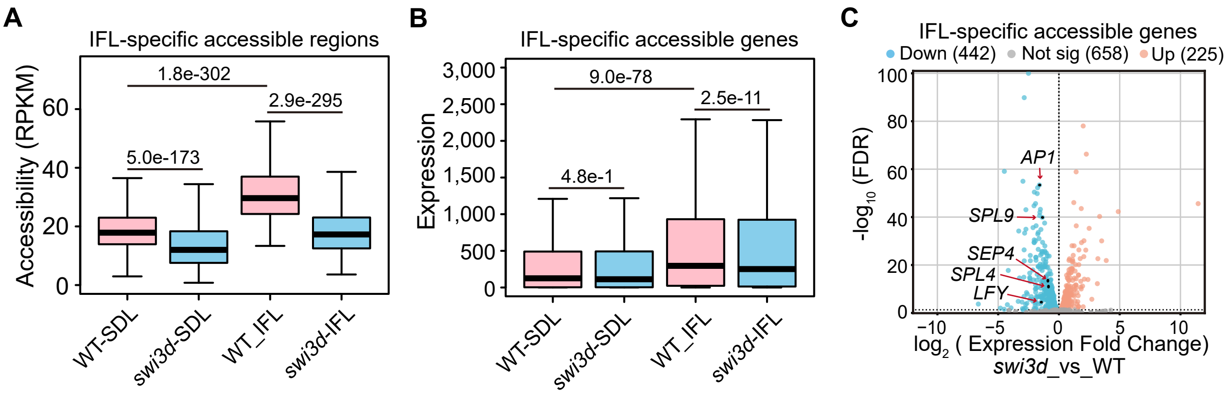

Supplementary Figure S7. Effects of *swi3d* mutation on accessibility and expression of inflorescence-specific accessible genes. (**A**) Boxplots showing the accessibility of inflorescence-specific accessible regions in seedlings and inflorescences of the wild type and *swi3d* mutant. (**B**) Boxplots displaying the expression levels of inflorescence-specific accessible genes in seedlings and inflorescences of the wild type and *swi3d* mutant. The center lines and box edges of the box plots represent medians and the interquartile range (IQR), respectively. Whiskers extend within 1.5 times the IQR. *P* values were determined by two-tailed Wilcoxon signed rank test. (**C**) Volcano plot showing the differential expression of inflorescence-specific accessible genes in the *swi3d* mutant compared to the wild type. The fold change and FDR were calculated using the inflorescence RNA-seq data. Genes that are up-regulated in *swi3d* (log_2(fold change)>0, FDR<0.05) are marked in red, genes that are down-regulated (log_2(fold change)<0, FDR<0.05) are marked in blue, and genes that are not significantly differentially expressed (FDR<0.05) are marked in grey. Representative points are marked with gene names.

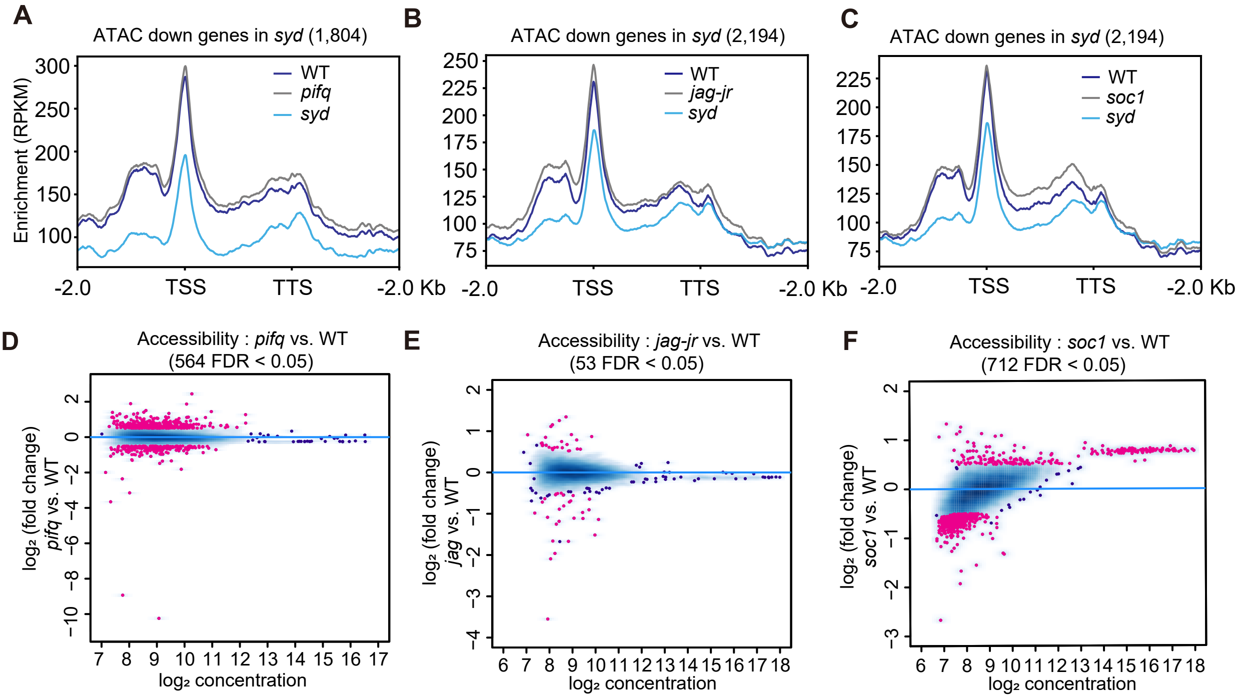

Supplementary Figure S8. The chromatin accessibility changes in transcription factor mutants. (**A-C**) Profile plots depicting the ATAC-seq signals of transcription factor mutants, *syd* and the wild-type plants at the genic regions with down-regulated accessibility in *syd*. (**D-F**) MA plots showing the differential analysis results of ATAC-seq peaks in transcription factor mutants versus the wild-type plants. The plot shows the $\log_2$ (fold change) and $\log_2$ (average reads concentration) of the ATAC-seq peaks. The red points represent the significant changed peaks with FDR<0.05. The ATAC-seq experiment in (**A, D**) was performed using 12-day-old seedlings and the experiments in (**B, C, E, F**) were performed using the inflorescences.

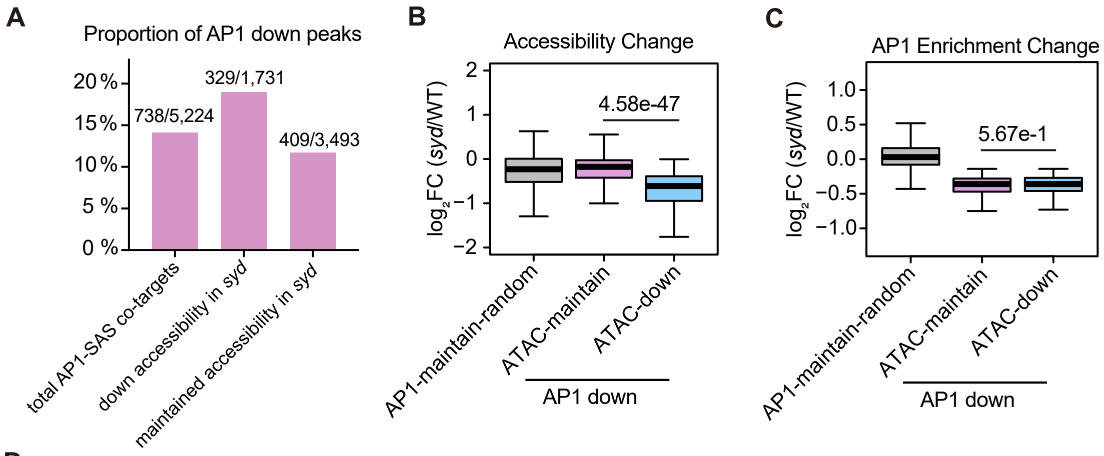

D

accession_number AGI	Protein	Type	mascot_score	mw	matched_queries
IPI00543034 AT1G69120	AP1	MADS_TF	900	30163	22
IPI00535368 AT1G18450	ARP4	SWI/SNF BAS/SAS/MAS	399	48905	10
IPI00522405 AT3G60830	ARP7	SWI/SNF BAS/SAS/MAS	306	39879	8
IPI00516255 AT1G24260	SEP3	MADS_TF	236	29048	5
IPI00539458 AT5G14170	SWP73B	SWI/SNF BAS/SAS/MAS	162	59233	4
IPI00541035 AT5G15800	SEP1	MADS_TF	141	28638	3
IPI00531522 AT2G45650	AGL6	MADS_TF	71	28783	1
IPI00529654 AT2G46020	BRM	SWI/SNF BAS	68	245317	2
IPI00528122 AT1G65480	FT	MADS_TF	63	19796	2
IPI00527426 AT2G45660	AGL20	MADS_TF	59	24518	2
IPI00525437 AT2G33610	SWI3B	SWI/SNF MAS	49	52378	1
IPI00522952 AT5G60910	AGL8	MADS_TF	49	27519	1
IPI00548531 AT5G20240	PI	MADS_TF	41	24031	1
IPI00536448 AT2G22540	SVP	MADS_TF	40	26880	1
IPI00517975 AT2G03710	SEP4	MADS_TF	33	21921	1
IPI00548723 AT3G22990	LFR	SWI/SNF SAS/MAS	32	50081	1
IPI00520479 AT1G32730	MIS	SWI/SNF MAS	20	36311	1

Supplementary Figure S9. Relationship between SAS loss and AP1 binding loss. (**A**) Proportion of the down-regulated AP1 binding regions among the total AP1-SAS co-binding targets (5,224), the AP1-SAS targets that show down-regulated accessibility in *syd* (1,731) and the AP1-SAS targets that show maintained accessibility in *syd* (3,493). (**B-C**) Boxplot showing the $\log_2$(fold change) of chromatin accessibility in *syd* relative to the wild-type (**B**) and the enrichment of AP1 (**C**) at random regions of AP1-SAS targets that show maintained AP1 enrichment (400), AP1-SAS targets that show maintained accessibility in *syd* (409), and AP1-SAS targets that show down-regulated accessibility in *syd* (329), respectively. In (**B**) and (**C**), the center lines and box edges of the box plots represents medians and the interquartile range (IQR), respectively. Whiskers extent within 1.5 times the IQR. *P* values were determined by two-tailed Mann-Whitney *U* test. (**D**) MADS transcription factors and SWI/SNF subunits identified in the AP-MS result of AP1 in inflorescences.
